# Hepatopathy in Scrub Typhus: Clinical Presentation, Association With Morbidity and Impact on Outcome

**DOI:** 10.7759/cureus.52316

**Published:** 2024-01-15

**Authors:** Rishabh Chauhan, Sohaib Ahmad, Chandan Goyal, Pavit Tewatia

**Affiliations:** 1 Internal Medicine, Himalayan Institute of Medical Sciences, Swami Rama Himalayan University, Dehradun, IND

**Keywords:** tropical fevers, renal failure, scrub typhus, hepatopathy, liver dysfunction

## Abstract

Introduction: Acute liver injury accompanies tropical fevers like scrub typhus. This study was undertaken to evaluate liver injury in scrub typhus and its association with the disease severity.

Methods: This was a single-centre prospective, observational study on in-patients of scrub typhus from north India. All patients were categorized on basis of elevation of transaminases as having normal or abnormal liver function. Those with hepatopathy were sub-categorized as having mild, moderate, severe or very severe liver injury.

Results: Liver dysfunction was present in 76/109 of the patients and was significantly associated with eschar, clinically discernible hepatomegaly and splenomegaly. Shock, renal and respiratory insufficiency, need for intensive care and oxygen supplementation were also significantly associated with hepatopathy. Duration of hospitalization and mortality were comparable in patients with/without liver injury; however delayed defervescence (6.2+3.8 vs. 4.5+2.5 days; p=0.025) was observed with hepatopathy. Icterus (p=0.001), hepatomegaly (p=0.015), thrombocytopenia (p<0.001) and raised erythrocyte sedimentation rate (ESR) (p=0.003) were significantly observed with increasing grade of liver injury.

Conclusion: Liver dysfunction and its increased severity in scrub typhus did not translate into increased morbidity and/or poor outcomes.

## Introduction

Scrub typhus is a life-threatening arthropod-borne febrile illness caused by the bacterium *Orientia tsutsugamushi* accounting for 11.2-14.4% of all cases presenting as acute febrile illnesses in north India [1-3]. Karp and Gilliam strains have been identified as the dominant strains in north India. The strains differ in their virulence as well as in their ability to produce an eschar [4]. *Orientia tsutsugamushi* evades the immune system and invades and multiplies in phagocytes. On getting released from the phagocytes, the organisms proliferate on the endothelium of the small blood vessels triggering the release of cytokines. This inflammatory response disrupts the endothelial integrity, causing fluid leakage, platelet aggregation, polymorphs and monocyte proliferation, leading to focal occlusive endarteritis causing microinfarcts. This process especially affects skeletal muscles, skin, lungs, kidneys, brain and cardiac muscles [5].

The patients usually present after a median period of eight days [3] as uncomplicated febrile illness or as mono- or multi-organ dysfunction culminating in a fatal outcome. As evidence of receiving appropriate treatment prior to hospitalization is usually lacking, it is not possible to ascribe adverse outcomes to delayed diagnosis and/or treatment.

Liver is frequently involved in scrub typhus manifesting mainly as elevation of transaminases and bilirubin. While these biochemical abnormalities are mainly non-specific, transaminitis observed in those with the infection may represent mild focal inflammation due to vasculitis of intrahepatic sinusoidal endothelium and cytopathic liver damage [6]. Few case reports observed bi- or triple nucleated changes and fat deposition in swollen hepatocytes and small lymphocyte aggregation in sinusoids [7]; granulomatous changes [8,9], and rod-shaped organisms within the hepatocytes and sinusoids with variable degrees of cytoplasmic organelle damage [10]. However, credible evidence clarifying the relationship between scrub typhus and hepatitis, clinical as well as histopathological, is lacking.

Focal death of individual hepatocytes causes hepatic injury without clinical liver failure. An established scoring system for scrub typhus utilizes liver related factors viz. serum albumin and aspartate transaminase levels as predictors of severity for scrub typhus [11] and we hypothesized that greater degree of liver dysfunction is associated with severe disease and poor outcome. We undertook this study to assess the liver functions in cases with scrub typhus and study the association of liver function with the severity of the disease in patients hospitalized with the disease.

## Materials and methods

This cross-sectional prospective observational study was carried out in accordance with the standards of the Helsinki Declaration at a tertiary referral center of Uttarakhand over a period of one year after obtaining institutional ethical clearance (vide letter no SRHU/HIMS/ETHICS/2022/279 dated 14/09/2022) and written informed consent from febrile patients aged 18 years or more with scrub typhus (positive immunoglobulin M (IgM) antibodies by Scrub Typhus Detect IgM ELISA by InBios International, Inc., Seattle, WA, USA). Excluded were those with tests positive for other tropical illnesses viz. dengue, malaria, leptospirosis, viral hepatitis and enteric fever. Also, those with history of pre-existing liver diseases were excluded. Those included were subjected to a detailed liver function test on admission (including alanine aminotransferase (ALT), aspartate aminotransferase (AST), alkaline phosphatase (ALP), total protein, albumin, bilirubin and international normalised ratio (INR)) besides the routine investigations needed for diagnosis and follow-up. The cases were categorised as having normal liver function (normal ALT and/or AST) or dysfunction graded as mild (less than two times), moderate (two to five times), severe (five to 10 times) or very severe (more than 10 times) elevation of any or both of the liver transaminases with or without hyperbilirubinemia. Details in clinical history (reported by the patients and attendants), physical examination, demographic parameters, laboratory parameters, complications and outcomes of individual patients were compiled.

Reporting of fever, seizures and/or bleeding was accepted (even if these weren’t witnessed or there was no documentation of the same); temperature was measured on admission and routinely thereafter in hospital. Defervescence was defined as abatement of fever for at least 24 hours without anti-pyretic administration. Hepatomegaly was considered if the liver span was >15 cm clinically or radiologically; palpable spleen or ultrasonographical enlargement was taken as splenomegaly. Severity/morbidity of the infection was considered on the basis of associated organ dysfunction, need for intensive care, dialysis, length of hospital stay (days) and mortality (improved or expired). Among the parameters of morbidity studied were severe anemia (hemoglobin <7 g/dl), thrombocytopenia (platelet count <150×103/cumm), hypotension/shock (systolic blood pressure <80 mmHg) and hyperbilirubinemia (bilirubin >3mg/dl). Renal insufficiency was defined as oliguria (output <400 ml/24 hours) and raised serum creatinine (>3 mg/dl) with no improvement with rehydration. Respiratory involvement (respiratory distress) was defined as tachypnea (>20/min) along with a fall in saturation of oxygen to <90%. Acute respiratory distress syndrome (ARDS) was defined as arterial oxygen partial pressure (PaO2)/fractional inspired oxygen (FiO2) <200 mmHg along with radiological appearance of bilateral non-homogenous opacities. Central nervous system involvement was considered if there was failure to localize or respond appropriately to noxious stimuli or if coma persisted for >30 min after generalized convulsion [12].

Statistical analysis was performed using the SPSS software 22 (IBM Corp., Armonk, NY, USA). Mean was the measure of central tendency and standard deviation the measure of dispersion for descriptive statistics. Association between categorical variables was assessed using the Chi-square/Fisher exact test (wherever applicable). Unpaired Student's T-test was used to assess the differences in the means between continuous variables. P-value <0.05 was taken as significant. Analysis of variance (ANOVA) was estimated on the means of the subcategories of liver dysfunction.

## Results

Of the 109 patients of scrub typhus (54% males), 44% were aged between 18-40 years. The main presenting features were fever (95%), loss of appetite (57%) and myalgia (48%); pallor (40%), hepatomegaly (27%) and eschar (17%) were the main examination findings. All patients were treated with doxycycline (100 mg twice a day) or azithromycin (500 mg once a day) for seven to 14 days by intravenous or oral route.

Liver dysfunction was seen in 70% (n=76) of patients followed by renal insufficiency (n=48; 44%) and respiratory distress (n=46; 42%). The main laboratory parameters were thrombocytopenia (120.8±78.9 x 103/cumm), INR (1.19±0.33), serum bilirubin (2.33±3.04 mg/dl), AST (223.37±184.3 IU/L), ALT (163.2±101.4 IU/L), ALP (285.06±211.31 IU/L), serum albumin (2.77±0.54 g/dl) and serum creatinine (1.72±1.60). Thirty-four percent (37/109) of patients required ICU stay of whom 43.2% (17/37) expired.

Oliguria, hepatomegaly, splenomegaly, and presence of eschar had a significant association with liver injury. Liver injury (n=76) was also significantly associated with severe anemia (p=0.009), packed cell transfusions (p=0.021), intensive care (p=0.045) and non-invasive oxygen administration (p=0.016); other organ dysfunctions were not significantly associated. Almost half the patients with liver dysfunction were hypoxemic (39/76) at presentation. Fifty-one percent each of patients with liver injury had renal (39/76) and respiratory insufficiency (39/76). Delayed defervescence (6.2+3.8 vs. 4.5+2.5 days; p=0.025) was observed with hepatopathy. 

Table 1 shows the comparison of various demographic, clinical, hematological and biochemical parameters, complications, and outcomes of scrub typhus in relation to the severity of liver injury. Icterus (p=0.001) and hepatomegaly (p=0.015), and thrombocytopenia (p<0.001) and raised ESR (p=0.003) were significantly observed with increasing grade of liver injury.

**Table 1:** Comparison of clinical, hematological and biochemical parameters, complications, and outcomes of scrub typhus with grading of severity of liver injury. TLC: total leucocyte count; ESR: erythrocyte sedimentation rate at 1 hour; INR: international normalized ratio; BUN: blood urea nitrogen; ALT: alanine transaminase; AST: aspartate transaminase; ALP: alkaline phosphatase; HD: hemodialysis; BIPAP: Bilevel positive airway pressure; ICU: intensive care unit; CNS: central nervous system

	No liver injury n=9	Mild liver injury n=10	Moderate liver injury n=31	Severe liver injury n=50	Very severe liver injury n= 9	p value
Clinical Features						
Fever	8 (88.8)	8 (80.0)	31 (100.0)	48 (96.0)	9 (100.0)	NS
Headache	4 (44.4)	1 (10.0)	12 (38.7)	19 (38.0)	2 (22.2)	NS
Vomiting	4 (44.4)	1 (10.0)	9 (29.0)	17 (34.0)	3 (33.3)	NS
Nausea	3 (33.0)	1 (10.0)	8 (25.8)	18 (36.0)	2 (22.2)	NS
Pain abdomen	3 (33.0)	2 (20.0)	13 (41.9)	21 (42.0)	2 (22.2)	NS
Diarrhea	1 (11.1)	2 (20.0)	1 (3.2)	4 (8.0)	0 (0)	NS
Myalgia	4 (44.4)	5 (50.0)	11 (35.4)	30 (60.0)	2 (22.2)	NS
Jaundice	0 (0)	0 (0)	4 (12.9)	`16 (32.0)	5 (55.5)	<0.001
Shortness of breath	1 (11.1)	4 (40.4)	15 (48.3)	18 (36.0)	1 (11.1)	NS
Conjunctival suffusion	0 (0)	3 (30.0)	1 (3.2)	8 (16.0)	1 (11.1)	NS
Decreased urine output	0 (0)	3 (30.0)	8 (25.8)	17 (34.0)	3 (33.3)	NS
Cough	0 (0)	2 (20.0)	8 (25.8)	6 (12.0)	0 (0)	NS
Arthralgia	2 (22.2)	1 (10.0)	5 (16.1)	8 (16.0)	2 (22.2)	NS
Loss of appetite	5 (55.5)	4 (40.0)	19 (61.2)	26 (52.0)	7 (77.7)	NS
Altered sensorium	2 (22.2)	1 (10.0)	6 (19.3)	11 ( 22.0)	3 (33.3)	NS
Hepatomegaly	0 (0)	0 (0)	9 (29.0)	16 (32.00	5 (55.5)	0.02
Splenomegaly	0 (0)	0 (0)	3 (9.6)	8 (16.0)	4 (44.4)	0.02
Eschar	1 (11.1)	0 (0)	5 (16.1)	13 (26.0)	0 (0)	NS
Pallor	4 (44.4)	5 (50.0)	10 (32.2)	22 (44.0)	3 (33.3)	NS
Icterus	2 (22.2)	0 (0)	4 (12.9)	22 (44.0)	6 (66.6)	<0.001
Bleeding	1 (11.1)	0 (0)	2 (6.4)	4 (8.0)	2 (22.2)	NS
Hematological parameters						
Hemoglobin (g/dl)	9.8 ± 2.9	5.8 ± 3.6	6.7 ± 2.5	8.3 ± 2.8	9.0 ± 1.6	NS
TLC (x 10^3^/cumm)	10.4 ± 5.7	10.8 ±7.3	10.6 ±4.9	10.7 ± 4.5	12.1 ± 10.0	NS
Platelet count (x 10^3^/cumm)	218.7 ± 79.5	149.1 ± 87.0	108.7 ± 62.5	116.5 ±77.4	57.5 ±27.5	<0.001
ESR (mm/hr)	10.3 ± 22.5	11.6 ± 25.7	11.9 ±20.5	10.4 ±8.8	11.2 ±4.4	0.003
INR	3.3 ± 0.0	2.6 ± 0.2	2.8 ± 0.2	2.6 ± 0.3	2.5 ± 0.6	NS
Biochemical parameters						
Potassium (mmol/l)	4.0 ± 0.3	4.0 ± 1.0	3.9 ± 0.7	4.0 ± 0.5	4.3 ± 0.5	NS
Creatinine (mg/dl)	4.0 ± 2.6	4.0 ± 1.4	3.9 ±1.5	4.0 ± 1.6	4.3 ± 0.8	NS
BUN (mg/dl)	21.0 ± 24.9	36.3 ± 36.9	37.1 ± 27.2	34.6 ± 24.9	41.5 ± 28.3	NS
Sodium (mmol/l)	134.0 ± 7.0	139.8 ± 7.7	134.5 ± 4.6	135.0 ± 4.9	134.7 ± 6.0	NS
Bilirubin (mg/dl)	0.7 ± 0.4	0.6 ± 0.2	1.2 ± 1.6	3.0 ±3.4	5.2 ±4.0	<0.001
ALT (IU/L)	23.2 ±11.4	39.2 ± 17.0	122.0 ±37.6	207.6 ±58.5	336.5 ±134.6	<0.001
AST (IU/L)	30.8 ± 6.2	54.6 ± 14.3	134.1 ± 37.8	264.1 ± 63.1	684.0 ± 267.3	<0.001
ALP (IU/L)	115.4 ± 62.6	117.5 ± 65.0	226.8 ±133.6	333.0 ± 201.3	574.7 ± 299.2	<0.001
Albumin (g/dl)	3.3 ± 0.7	2.6 ± 0.7	2.8 ± 0.4	2.6 ± 0.4	2.5 ± 0.4	0.02
Outcome Measures						
Duration of hospitalization (days)	9.8 ± 6.2	5.8 ± 2.8	6.7 ± 3.0	8.3 ± 5.1	9.0 ± 4.8	NS
Defervescence (days)	5.3 ± 2.9	3.7 ± 2.4	5.1 ± 2.4	6.1 ± 3.1	6.7 ± 4.1	NS
Duration of symptoms (days)	6.4 ± 5.6	5.8 ± 3.6	6.8 ± 5.1	6.1 ± 3.6	7.6 ± 2.7	NS
Abnormal chest radiogram	2 (22.2)	4 (40.0)	10 (32.2)	25 (50.0)	5 (55.5)	NS
Respiratory Failure	5 (55.5)	7 (70.0)	13 (41.9)	23 (46.0)	4 (44.4)	NS
Anemia	3 (33.3)	4 (40.0)	13 (41.9)	20 (40.0)	2 (22.2)	NS
ICU Requirement	2 (22.2)	3 (30.0)	10 (32.2)	20 (40.0)	2 (22.2)	NS
Oxygen Requirement	4 (44.4)	8 (80.0)	14 (45.1)	30 (60.0)	6 (66.6)	NS
Mechanical Ventilation	0 (0)	2 (20.0)	2 (6.4)	12 (24.0)	1 (11.1)	NS
BIPAP	0 (0)	2 (20.0)	6 (19.3)	12 (24.0)	2 (22.2)	NS
Hemodialysis	0 (0)	1 (10.0)	2 (6.4)	2 (4.0)	0 (0)	NS
Packed RBC transfusion	1 (11.1)	3 (30.0)	2 (6.4)	7 (14.0)	1 (11.1)	NS
CNS involvement	2 (22.2)	1 (10.0)	6 (19.3)	11 (22.0)	333.3)	NS
Renal Insufficiency	2 (22.2)	3 (30.0)	16 (51.6)	23 (4.0)	4 (44.4)	NS
Shock	1 (11.1)	0 (0)	9 (29.0)	19 (38.0)	3 (33.3)	NS
Expired	0 (0)	4 (40.0)	3 (9.6)	7 (14.0)	2 (22.2)	NS

## Discussion

Nearly two-thirds of patients with scrub typhus had liver involvement at initial presentation. Ogawa et al. [13] also observed liver dysfunction in 76% of patients. Liver dysfunction comprised of transaminitis and reduced synthesis of the clotting factors and albumin as exemplified by raised prothrombin time and low albumin. Liver dysfunction is therefore a common manifestation in scrub typhus. In treatment-naïve patients, interferon gamma-induced activation of cell-mediated immune response and cytotoxic lymphocytes occurs as evidenced by elevated plasma concentrations of granzymes A and B, protein 10 and monokine [10,14]. Microscopically, while the architecture remains intact, hepatocytes swell with hyperplasia of Kupffer cells and creeping fibrosis from the portal area. Rickettsial organisms may also be observed in the cytoplasm of hepatocytes. With treatment, hepatocytes undergo balloon degeneration and portal tracts are infiltrated with mononuclear cells in small clusters within hepatic lobules; while Kupffer cell hyperplasia may persist, Rickettsiae disappear. Cholestasis or apoptosis of hepatocytes is usually absent [15].

The laboratory-proven coagulopathy seems to be partial or limited as it did not translate into bleeding events. Likewise, low albumin levels translated into fluid transudation in the extremities in a few patients and resolved in most of them on follow-up. The transaminitis was associated with prolongation of hospital stay with increasing severity of transaminitis; however, the same was not statistically significant. 

Renal insufficiency was significantly associated with liver dysfunction; however, the association was not significant with increasing severity of transaminitis. The difference in the mean serum creatinine and blood urea nitrogen between those with and without liver dysfunction was not statistically significant. Besides direct damage by the bacteria, renal insufficiency may be caused by possible mechanisms (prerenal failure, shock, rhabdomyolysis, vasculitis, acute interstitial nephritis, thrombotic microangiopathy) in isolation or combination [16]. Liver dysfunction was also significantly associated with shock and need for intensive care. Whereas the majority of those with shock at presentation had moderate, severe or very severe liver injury exemplified by transaminase elevation, the association was not significant statistically. The lack of association of the increasing transaminitis and organ dysfunctions reflects the presumably different mechanisms and differential affection of the various organs by the bacteria. It also reflects the organ/tissue-specific tropism by Orientia. The severity of thrombocytopenia and the duration of hospitalization significantly increased with the increasing severity of liver injury. In all the survivors, the elevated transaminases normalised on discharge or by the second week when they were followed up.

The major strength of the study lies in the fact that all patients were investigated thoroughly and standard management protocols were followed universally to reduce the analysis bias. The other causes of hepatitis were excluded in all the included patients. The limitations of the present study were the small sample size and that it was a hospital-based study. Therefore the study population may not represent the actual burden in the community and the results may not be extrapolated to the community. The duration of the symptoms and administration of drugs (especially acetaminophen) prior to testing the liver function was variable and may have affected the intensity of the transaminitis, the basis of grading of hepatopathy in the present study, by inducing liver microsomal enzymes. Moreover, the impact of alcoholic hepatitis on the liver transaminases could not be elucidated. Lastly, the molecular characterization of the organism was not performed which deprived us of understanding the virulence of the organ dysfunction. 

## Conclusions

Liver dysfunction, commonly associated with scrub typhus, seems to be merely a transaminitis which may be associated with other organ dysfunctions but is devoid of any impact on the outcome of the disease.
